# Increased Healthcare Service Utilizations for Patients with Dementia: A Population-Based Study

**DOI:** 10.1371/journal.pone.0105789

**Published:** 2014-08-26

**Authors:** Shiu-Dong Chung, Shih-Ping Liu, Jau-Jiuan Sheu, Ching-Chun Lin, Herng-Ching Lin, Chao-Hung Chen

**Affiliations:** 1 Division of Urology, Department of Surgery, Far Eastern Memorial Hospital, New Taipei City, Taiwan; 2 School of Medicine, College of Medicine, Fu Jen Catholic University, Taipei, Taiwan; 3 Sleep Research Center, Taipei Medical University Hospital, Taipei, Taiwan; 4 Department of Urology, National Taiwan University Hospital and College of Medicine, Taipei, Taiwan; 5 Department of Neurology, Taipei Medical University Hospital, Taipei, Taiwan; 6 Neuroscience Research Center, Taipei Medical University Hospital, Taipei, Taiwan; 7 Graduate Institute of Biomedical Informatics, College of Medical Science and Technology, Taipei Medical University, Taipei, Taiwan; 8 School of Health Care Administration Taipei Medical University, Taipei, Taiwan; 9 Department of Thoracic Surgery, Mackay Memorial Hospital, Taipei, Taiwan; 10 Mackay Junior College of Medicine, Nursing, and Management, Taipei, Taiwan; Institute of Psychiatry, United Kingdom

## Abstract

**Background:**

The majority of previous studies investigating the health care utilization of people with dementia were conducted in Western societies. There is little information on the economic burden on the healthcare system attributable to dementia in Asian countries. This study thus investigated differences in utilization of healthcare services between subjects with and those without a diagnosis of dementia using Taiwan’s National Health Insurance population-based database.

**Methods:**

This study comprised 5,666 subjects with a dementia diagnosis and 5,666 age- and gender-matched comparison subjects without a dementia diagnosis. We individually followed each subject for a 1-year period starting from their index date to evaluate their healthcare resource utilization. Healthcare resource utilization included the number of outpatient visits and inpatient days, and the mean costs of outpatient and inpatient treatments. In addition, we divided healthcare resource utilization into psychiatric and non-psychiatric services.

**Results:**

As for utilization of psychiatric services, subjects with a dementia diagnosis had significantly more outpatient visits (2.2 vs. 0.3, *p*<0.001) and significantly higher outpatient costs (US$124 vs. US$16, *p*<0.001) than comparison subjects. For non-psychiatric services, subjects with a dementia diagnosis also had significantly more outpatient visits (34.4 vs. 31.6, *p*<0.001) and significantly higher outpatient costs (US$1754 vs. US$1322, *p*<0.001) than comparison subjects. For all healthcare services, subjects with a dementia diagnosis had significantly more outpatient visits (36.7 vs. 32.0, *p*<0.001) and significantly higher outpatient costs (US$1878 vs. US$1338, *p*<0.001) than comparison subjects. Furthermore, the total cost was about 2-fold greater for subjects with a dementia diagnosis than for comparison subjects (US$3997 vs. US$2409, *p*<0.001).

**Conclusions:**

We concluded that subjects who had received a clinical dementia diagnosis had significantly higher utilization of all healthcare services than comparison subjects.

## Introduction

Dementia is a chronic disease in advanced age and is characterized by progressive cognitive decline that interferes with independent functioning, ultimately resulting in the need for long-term care [1], [2]. Previous studies established its prevalence as 2%∼10% of those over 65 years of age, and the incidence of dementia disorders increases exponentially beyond 65 years of age [3]–[8]. Dementia also incurs a significant economic burden of direct healthcare costs and non-healthcare costs [5], [9], [10].

Several studies reported the monetary impact of dementia in different populations [9]–[17]. For example, a study by Kane and Atherly found that dementia was associated with 2.4-times greater Medicare Part A (inpatient) payments and 1.3 times greater Part B (outpatient) payments for community-dwelling persons in the US [13]. Another study by Arling et al. in the US also reported that dementia combined with heavy nursing home (NH) use resulted in 2.5-fold greater Medicare and 12.6-fold greater Medicaid annual payments compared to a trajectory of dementia with little of no NH use [14]. Furthermore, one study stratified by care levels from German insurance claims data found that institutional care for dementia patients required additional yearly per capita expenses of ca. €200 on health and ca. €11,200 on long-term care [6]. Another study in Spain showed that mean numbers of patient visits for follow-up were 8.8 and 14.7 visits/semester to the primary care center for patients with Alzheimer’s disease (AD) and those with vascular dementia (VD), respectively, and medical costs per patient per semester were €2866 for AD and €3209 for VD patients [10].

While some studies reported the healthcare utilization of patients with dementia alone [10]–[17], others have attempted to compare differences in healthcare utilization or costs between patients with and those without dementia [9], [18]–[20]. For example, a study by Fick et al. found that the annual costs were US$7557 and US$4766 for the dementia-only group and neither dementia nor delirium group, respectively, in the US [9]. In the analyses of 2006 health insurance claims data in Germany, Schwarzkopf et al. reported that the annual expenditure within the social security system was about €12,300 per dementia patient and about €4,000 per non-demented control subject [18].

However, all such studies investigating the health care utilization of people with dementia were conducted in Western societies, and there is little information on the economic burden on the healthcare system attributable to dementia in Asian countries. In addition, almost no study attempted to differentiate health care service utilization due to psychiatric reasons and general health care service utilization. Therefore, the aim of this study was to investigate differences in the utilization of healthcare services between subjects with and those without a dementia diagnosis using Taiwan’s National Health Insurance (NHI) population-based database. Utilization of psychiatric and non-psychiatric services would further be examined separately.

## Methods

### Database

The sampled subjects were retrieved from the Taiwan Longitudinal Health Insurance Database (LHID2000). The LHID2000 consists of data on medical claims for a selected 1 million enrollees since the beginning of the Taiwan NHI program in 1995. The Taiwan National Health Research Institute and some researchers demonstrated the representativeness of the LHID2000 relative to the entire enrollees under the NHI program [21], [22] To date, there have been several hundred studies published in international peer-reviewed journals utilizing data from the NHI program [23]. The LHID2000, which was open to the researchers in Taiwan, was available from the NHRI (http://nhird.nhri.org.tw/date_01.html). This study is based on de-identified secondary data from the LHID2000 released by the NHRI without restrictions for research purposes.

This study was exempt from full review by the Institutional Review Board of Taipei Medical University because the LHID2000 consists of de-identified secondary data released to the public for research purposes.

### Study Sample

This study included a study group and a comparison group. For the study group, we first identified 6953 subjects who had received a diagnosis of dementia (ICD-9-CM code 290.0∼290.4, 294.1, 331.0∼331.2, and 331.82) in ambulatory care visits (including outpatient departments of hospitals and clinics) between January 1 and December 31, 2010. We then excluded those subjects aged <44 years (*n* = 65) in accordance with a prior study [24]. We also excluded those who died in 2010 and 2011 (*n* = 638) in order to assure equal follow-up periods (1 year) for all selected subjects. In addition, we excluded those who had a history of major psychosis or a substance-related disorder (ICD-9-CM codes 291∼299 or 303∼305), stroke (ICD-9-CM codes 430∼438), or traumatic brain injury (ICD-9-CM codes 801∼804 or 850∼854) prior to the index date (*n* = 584). Ultimately, 5,666 subjects with a dementia diagnosis were included in the study group. We further defined the date of their first visit for treatment of dementia in 2010 as the index date.

We also retrieved comparison subjects from the LHID2000. We likewise excluded all enrollees aged <44 years and those subjects who had ever received a diagnosis of dementia since initiation of the Taiwan NHI in 1995. We also excluded those who died in 2010 and 2011 and those with a diagnosed history of major psychosis, a substance-related disorder, stroke, or traumatic brain injury prior to the index date. Thereafter, we further selected 5,666 comparison subjects (1 comparison subjects per subject with dementia) from the remaining enrollees matched with study subjects by gender and age using the SAS proc surveyselect program (SAS, Cary NC, USA). Specifically, in each gender- and age-matched strata, a random sampling process was applied for the selection of comparison subjects. For comparison subjects, we defined the date of their first visit to a physician occurring during 2010 as the index date. Although comparison subjects might have to experience certain health problem for physician visits, by definition, in order to be present in the dataset, representativeness might not be a serious concern. Due to the very low out-of-pocket copayments, comprehensive benefits, unrestricted access to any medical institution of the patient’s choice, and a wide variety of providers including primary care physicians in Taiwan’s NHI program, people in Taiwan did visit the physicians and outpatient departments frequently. In the analyses of NHI program in 2002, only 7.7% persons did not have any visit [25]. The percentage might even be lower among elder population considered in our study.

In total, this study comprised a 11,332-person study sample including 5,666 subjects with a dementia diagnosis and 5,666 comparison subjects. We individually traced each subject for a 1-year period starting from their index date to estimate their healthcare resource utilization.

### Variables of interest

The variables of the healthcare resource utilization were defined as follows: number of outpatient visits and inpatient days and the mean costs of outpatient and inpatient treatments Furthermore, we divided healthcare resource utilization into psychiatric and non-psychiatric services.

### Statistical Analysis

We used the SAS statistical package (SAS System for Windows, vers. 8.2) to perform the statistical analyses in this study. We calculated the mean and standard deviation (SD) for all variables of the healthcare resource utilization. We performed Student’s *t*-tests to compare differences in variables of the healthcare resource utilization between subjects with and those without a dementia diagnosis. We further performed a multivariate regression analysis to model the logarithm of the mean costs as a linear function of a set of independent variables. Differences were considered significant for two-sided *p* values of ≤0.05.

## Results

Of the 11,332-person study sample, the mean age was 77.9 years with an SD of 9.3 years, and more than 70% were aged ≥75 years. Table 1 also shows that after matching for gender and age, there was no significant difference in urbanization level or geographic region between subjects with and those without a dementia diagnosis. However, subjects with a dementia diagnosis were more likely to have monthly income of ≤$US530 than comparison subjects (*p*<0.001).

**Table 1:** Demographic characteristics of subjects with a dementia diagnosis and comparison subjects (*n* = 11,332).

Variable	Subjects with a dementiadiagnosis (*n* = 5,666)		Comparison subjects(*n* = 5,666)		*p* value
	Total no.	Percent (%)	Total no.	Percent (%)	
Gender					1.000
Male	2486	43.9	2486	43.9	
Female	3180	56.1	3180	56.1	
Urbanization level					0.663
1 (most urbanized)	1407	24.8	1374	24.3	
2	1368	24.1	1372	24.2	
3	830	14.7	872	15.4	
4	1001	17.7	964	17.0	
5 (least urbanized)	1060	18.7	1084	19.1	
Monthly income (US$)					<0.001
$1∼530	3777	66.6	3401	60.0	
$530∼830	1681	29.7	1975	34.9	
≥$830	208	3.7	290	5.1	
Geographic region					0.948
Northern	2424	42.8	2410	42.5	
Central	1452	25.6	1479	26.1	
Southern	1613	28.5	1604	28.3	
Eastern	177	3.1	173	3.1	

Table 2 shows the healthcare resource utilization within the 1-year period following the index date for subjects with and those without a dementia diagnosis. As for utilization of psychiatric services, subjects with a dementia diagnosis had significantly more outpatient visits (2.22 vs. 0.34, *p*<0.001) and significantly higher outpatient costs (US$124 vs. US$16, *p*<0.001) compared to subjects without a dementia diagnosis. This implies that the mean cost of yearly outpatient visits for psychiatric services within the 1-year follow-up period was about 8-fold greater for subjects with a dementia diagnosis than subjects without a diagnosis of dementia.

**Table 2:** Use and costs (US$) of healthcare services within 1 year by subjects with a dementia diagnosis and comparison subjects.

Variable	Subjects with a dementiadiagnosis (*n* = 5666)		Comparison subjects(*n* = 5666)		*p* value
	Mean	SD	Mean	SD	
Psychiatric services					
Outpatients services (no. of visits)	2.22	4.76	0.34	1.96	<0.001
Outpatient costs (US$)	124	334	16	144	<0.001
Inpatient days	0.47	7.25	0.05	2.04	<0.001
Inpatient costs (US$)	46	638	4	122	<0.001
Total costs (US$)	170	752	20	211	<0.001
Non-psychiatric services					
Outpatients services (no. of visits)	34.43	25.27	31.63	23.24	<0.001
Outpatient costs (US$)	1754	2829	1322	2433	<0.001
Inpatient days	10.25	27.08	4.63	17.65	0.019
Inpatient costs (US$)	2073	5703	1067	4017	<0.001
Total costs (US$)	3827	6621	2389	5037	<0.001
All health services					
Outpatients services (no. of visits)	36.65	26.01	31.97	23.47	<0.001
Outpatient costs (US$)	1878	2843	1338	2482	<0.001
Inpatient days	10.71	27.99	4.68	17.79	<0.001
Inpatient costs (US$)	2119	5732	1071	4019	<0.001
Total costs (US$)	3997	6631	2409	5034	<0.001

SD, standard deviation.

As to utilization of non-psychiatric services, Table 2 shows that subjects with a dementia diagnosis also had significantly more outpatient visits (34.43 vs. 31.63, *p*<0.001) and significantly higher outpatient costs (US$1754 vs. US$1322, *p*<0.001) than comparison subjects. Furthermore, inpatient costs for subjects with a dementia diagnosis were almost 2-fold greater compared to comparison subjects (US$2073 vs. US$1067, *p*<0.001).

Table 2 also shows the use and costs of all healthcare services. Subjects with a dementia diagnosis had significantly more outpatient visits (36.65 vs. 31.97, *p*<0.001) and significantly higher outpatient (US$1878 vs. US$1338, *p*<0.001) and total (US$3997 vs. US$2409, *p*<0.001) costs than subjects without a dementia diagnosis. This indicates that the total cost was about 1.67-fold greater for subjects with a dementia diagnosis than subjects without a diagnosis of dementia.

Table 3 shows the adjusted relationship between total costs for all healthcare services and dementia. Multiple regression analyses revealed that subjects with a dementia diagnosis had significantly higher total costs (*p*<0.001) for all healthcare services compared to subjects without a diagnosis of dementia after adjusting for urbanization level, monthly income, and geographic region.

**Table 3:** Multiple regression analysis of adjusted relationships between log costs of all health services and dementia.

Variable	Log (all health services costs)	
	B	*p* value
Group		
Subjects with a dementia diagnosis	0.679	<0.001
Comparison subjects		
Urbanization level		
1 (most urbanized)		
2	−0.036	0.323
3	−0.057	0.183
4	−0.083	0.061
5 (least urbanized)	−0.069	0.124
Monthly income (US$)		
$1∼530	−0.208	<0.001
$530∼830	−0.535	<0.001
≥$830		
Geographic region		
Northern		
Central	0.012	0.718
Southern	0.081	0.013
Eastern	0.210	0.005

## Discussion

This study compared differences in the utilization of healthcare services between subjects with a dementia diagnosis and gender- and aged-matched subjects without a diagnosis of dementia. As for the utilization of psychiatric services, we found that the mean number of yearly outpatient visits and total costs for psychiatric services within the 1-year follow-up period were 7- (2.2 vs. 0.3) and 8.5-fold (US$170 vs. US$20) greater, respectively, for subjects with a dementia diagnosis compared to comparison subjects. As to the utilization and costs of non-psychiatric services, subjects with a dementia diagnosis had significantly more outpatient visits (34.4 vs. 31.6) and higher total costs than comparison subjects (US$3827 vs. US$2389). Moreover, subjects with a dementia diagnosis had a significantly higher total cost for all health services compared to comparison subjects (US$3997 vs. US$2409).

Dementia is a growing public health problem [9]. Several studies reported that dementia alone accounts for billions of dollars in healthcare-resource utilization [10]–[17], [26]. Previous studies even showed that annual costs of dementia ranged US$3927∼US$56,290 for patients with dementia in different populations and periods [6], [9], [10], [15]–[17]. In further comparison of differences in utilization between patients with and those without dementia, excess healthcare costs and utilization were reported for patients with a diagnosis of dementia [18]–[20]. For instance, in the US, Fick et al. compared differences in total costs and outpatient visits between subjects with and those without dementia [9]. They found that the total costs were $7557 and $4766 for subjects with and those without dementia, respectively. In other words, it indicated that the total costs were 1.6-times greater for subjects with dementia than for comparison subjects. Their result was very similar to that reported in our study (1.67-times).

Furthermore, we compared utilization and costs of psychiatric, non-psychiatric, and all health services within the 1-year follow-up period among subjects with a dementia diagnosis and comparison subjects in this study. We found that subjects with a dementia diagnosis had greater utilization of psychiatric services in terms of outpatient visits and total costs (2.2 vs. 0.3 and US$170 vs. US$20, respectively) and also had greater utilization of non-psychiatric services in terms of outpatient visits and total costs (34.4 vs. 31.6 and US$3827 vs. US$2389, respectively) than comparison subjects during the 1-year follow-up. An explanation might be because patients with dementia are more likely to have more coexisting chronic health problems than those without dementia. One study confirmed that certain chronic diseases (e.g., stroke, diabetes, hypertension, heart disease, lung disease, cancer, and psychiatric problems) were more common in persons with dementia than in the general population [27].

Our study used a nationwide population-based dataset to mitigate the effect of selection biases and provide a sufficient sample size to detect differences in healthcare service utilization between subjects with a dementia diagnosis and comparison subjects. However, limitations of this study should be noted. First, diagnoses of dementia were identified from the administrative database through ICD-9-CM codes reported by physicians and released by the Bureau of the NHI. Therefore, we assured that all selected dementia cases were diagnosed by a certified psychiatrist to increase the validity of the dementia diagnosis. Second, our study focused on people with dementia who had received a diagnosis from clinical services. Indeed, some people with dementia had not been diagnosed. And those with a diagnosis might differ from the unrecognized cases, as a clinical diagnosis might itself affect access to services. Third, we did not evaluate indirect costs associated with dementia such as missed work days, time in a nursing home, or formal and informal care in this study. Finally, the claims data lack information on the cognitive status, and the severity of dementia could not be determined. Therefore, we were unable to evaluate the utilization of healthcare services according to dementia severity.

Despite these limitations, this study found that subjects with a dementia diagnosis had significantly higher utilization of all healthcare services than comparison subjects. Accurately identifying costs attributable to dementia is a challenge. Future studies are encouraged to estimate the total costs including missed work days, time in a nursing home, and formal and informal care attributable to dementia and investigate whether more-accurate and earlier treatment could reduce healthcare costs related to medical comorbidities.
